# New Antibody-Free Mass Spectrometry-Based Quantification Reveals That C9ORF72 Long Protein Isoform Is Reduced in the Frontal Cortex of Hexanucleotide-Repeat Expansion Carriers

**DOI:** 10.3389/fnins.2018.00589

**Published:** 2018-08-28

**Authors:** Arthur Viodé, Clémence Fournier, Agnès Camuzat, François Fenaille, NeuroCEB Brain Bank, Franck Letournel, Morwena Latouche, Fanny Elahi, Isabelle Le Ber, Christophe Junot, Foudil Lamari, Vincent Anquetil, François Becher

**Affiliations:** ^1^Service de Pharmacologie et Immunoanalyse, Laboratoire d’Etude du Métabolisme des Médicaments, Commissariat à l’Énergie Atomique et aux Énergies Alternatives, Institut National de la Recherche Agronomique, Université Paris Saclay, Gif-sur-Yvette, France; ^2^Inserm U 1127, CNRS UMR 7225, Institut du Cerveau et de la Moelle Èpinière, ICM, Sorbonne Université, Paris, France; ^3^Assistance Publique – Hôpitaux de Paris, Hôpital Pitié-Salpêtrière, Paris, France; ^4^Ecole Pratique des Hautes Etudes, PSL Research University, Paris, France; ^5^Department of Neurology, Memory and Aging Center, University of California, San Francisco, San Francisco, CA, United States; ^6^National Reference Center for Rare or Early Dementias, Institute of Memory and Alzheimer’s Disease (IM2A), Department of Neurology, AP-HP – Hôpital Pitié-Salpêtrière, Paris, France; ^7^Assistance Publique – Hôpitaux de Paris, Service de Biochimie Métabolique, Hôpitaux Universitaires Pitié Salpêtrière – Charles Foix, Paris, France; ^8^GRC 13 Neurométabolisme – UPMC, Sorbonne Université, Paris, France

**Keywords:** frontotemporal dementia (FTD), frontotemporal lobar degeneration (FTLD), amyotrophic lateral sclerosis (ALS), C9ORF72, TDP-43, TDP43, mass spectrometry (MS), GRN

## Abstract

Frontotemporal dementia (FTD) is a fatal neurodegenerative disease characterized by behavioral and language disorders. The main genetic cause of FTD is an intronic hexanucleotide repeat expansion (G_4_C_2_)n in the *C9ORF72* gene. A loss of function of the C9ORF72 protein associated with the allele-specific reduction of *C9ORF72* expression is postulated to contribute to the disease pathogenesis. To better understand the contribution of the loss of function to the disease mechanism, we need to determine precisely the level of reduction in C9ORF72 long and short isoforms in brain tissue from patients with *C9ORF72* mutations. In this study, we developed a sensitive and robust mass spectrometry (MS) method for quantifying C9ORF72 isoform levels in human brain tissue without requiring antibody or affinity reagent. An optimized workflow based on surfactant-aided protein extraction and pellet digestion was established for optimal recovery of the two isoforms in brain samples. Signature peptides, common or specific to the isoforms, were targeted in brain extracts by multiplex MS through the parallel reaction monitoring mode on a Quadrupole–Orbitrap high resolution mass spectrometer. The assay was successfully validated and subsequently applied to frontal cortex brain samples from a cohort of FTD patients with *C9ORF72* mutations and neurologically normal controls without mutations. We showed that the C9ORF72 short isoform in the frontal cortices is below detection threshold in all tested individuals and the C9ORF72 long isoform is significantly decreased in *C9ORF72* mutation carriers.

## Introduction

Frontotemporal dementia (FTD) is the second most prevalent neurodegenerative disease before the age of 65, after Alzheimer disease. FTD are caused by frontal and temporal lobar degeneration leading to behavioral, socioemotional, language disorders, and progressive loss of autonomy and death approximately 10 years after disease onset (Rascovsky et al., 2011). Amyotrophic lateral sclerosis (ALS) caused by motor neuron degeneration is associated with FTD in 15% of patients or families. Familial forms of FTD, accounting for about 20–50% of cases (Rosso, 2003) are mainly caused by mutations in three major genes: granulin (*GRN*), microtubule-associated protein tau (*MAPT*) and C9 open reading frame 72 (*C9ORF72*).

*C9ORF72*, the most frequent genetic etiology, represents 25% of familial FTD cases (DeJesus-Hernandez et al., 2011; Renton et al., 2011), 80% of FTD-ALS familial cases and also explains 40% of pure familial ALS (without FTD) (Majounie et al., 2012). An expansion of hundreds to thousands hexanucleotide (G_4_C_2_) repeats is present in the first intron of the gene in patients, while healthy individuals carry less than 24 (G_4_C_2_) repeats. While the exact mechanisms of disease remain unknown, two main modes of toxicity are proposed. The expansion may be deleterious through formation of nuclear RNA foci by RNA containing the hexanucleotide expansion (G_4_C_2_) with sequestration of RNA-binding proteins (Lagier-Tourenne et al., 2013), and/or translation of polydipeptides proteins that aggregate in the brain (Mori et al., 2013). A loss of function of the C9ORF72 protein associated with a decrease in *C9ORF72* allele-specific expression was also hypothesized to contribute to the pathogenesis based on reduction of *C9ORF72* mRNA transcript levels in patients (Ciura et al., 2013). The three alternatively spliced *C9ORF72* transcripts encode two C9ORF72 protein isoforms, a 222 amino acids (AA) protein isoform called C9-short (C9-S) and a 481AA protein isoform called C9-long (C9-L). The two protein isoforms have been shown by immunofluorescence to have distinct cellular localization with the C9-S lozalizing to the nuclear membrane and C9-L to the cytoplasm (Xiao et al., 2016). This observation suggests that the two isoforms have a different function, while the precise function of the protein has not been clearly determined. A potential role in endosomal transport and autophagy was reported through interaction with Rab-GTPases (Zhang et al., 2012; Levine et al., 2013).

A better understanding of the contributions of the loss of function to the disease mechanism requires a precise quantification of reduction in levels of C9ORF72 isoforms. C9ORF72 protein has been so far only quantified in human tissues by Western blot (Waite et al., 2014). Several studies pointed out the poor affinity and selectivity of commercially available antibodies (Waite et al., 2014; Davidson et al., 2018), requiring laborious in-house generation of antibodies. Targeted mass spectrometry (MS) is a powerful alternative to Western blot and enzyme-linked immunosorbent assay (ELISA) for quantification of proteins. It provides accurate quantification, high level of specificity, avoiding interference due to cross-reactivity of antibodies, and the ability to discriminate between isoforms (Chen D. et al., 2015; Chen Y.-T. et al., 2015; Jedrychowski et al., 2015; Lesur et al., 2015). Nevertheless, MS-based detection of low-abundant proteins in complex fluids or tissues remains challenging without efficient sample preparation protocols. The gold standard relies on the combination with immunoprecipitation to selectively enrich the analyte of interest prior to MS (Chen Y.-T. et al., 2015), but is applicable only when antibodies with sufficient specificity and affinity for the target protein are available.

We have developed a sensitive and robust antibody-free MS assay for quantification of C9ORF72 isoforms in brain samples. The protocol consists in an optimized tissue lysis protocol followed by pellet digestion of extracted brain proteins and specific monitoring of common and isoform specific peptides by targeted high-resolution MS in the parallel reaction monitoring mode (PRM). Reproducibility and linearity were demonstrated, as well as equivalent isoform recovery from brain tissue samples and stability during sample preparation. This new assay allowed for the first time the quantification of the C9ORF72 long isoform in *post mortem* frontal cortex brain samples from a cohort of FTD patients harboring a *C9ORF72* mutation and highlighted a significant decrease in concentrations in mutation carriers. The short isoform was found to be below the sensitivity threshold of the method.

## Materials and Methods

### Patients Information, Tissue Collection, and Consent

Frozen tissue from frontal cortex (Brodmann area 9/10) of 21 FTD (with or without secondarily developed ALS) patients carrying *C9ORF72* expansion, of 10 patients with non-genetic FTD (with or without secondarily developed ALS) pathologically characterized by TDP-43-positive neuronal inclusions, and of 12 neurologically healthy controls were studied. The brain samples were collected through a brain donation program dedicated to neurodegenerative dementias coordinated by the NeuroCEB Brain Bank Network. The informed consent for post-mortem examination and research studies was signed by the legal representative of each patient in patient’s name, as allowed by the French law and approved by the local ethics committee and the brain bank has been officially authorized to provide samples to scientists (agreement AC-2013-1887). All procedures performed in this study involving human participants were in accordance with the ethical standards of the institutional research committees and with the 1964 Helsinki declaration. The brain banks fulfill criteria from the French Law on biological resources including informed consent, ethics review committee and data protection (article L1243-4 du Code de la Santé publique, August 2007). The Neuro-CEB brain bank (BioResource Research Impact Factor number BB-0033-00011) has been declared to the Ministry of Research and Higher Education, as required by French law.

### Chemical and Materials

C9ORF72 short isoform was purchased from Proteintech (cat# ag21080) (Proteintech Group, Chicago, IL, United States) and C9ORF72 long isoform from Abnova (cat#00203228-P01) (Abnova Le Perray En Yvelines, France). Trypsin from bovine pancreas TPCK Treated (reference T1426), ammonium hydroxide, ammonium bicarbonate were purchased from Sigma-Aldrich (Saint Quentin Fallavier, France). RapiGest SF Surfactant and SPE Oasis Max 1CC/30 mg were purchased from Waters Corporation (Milford, MA United States). Labeled peptides for quantification were synthesized in Absolute QUAntitation (AQUA) ultimate quality by Thermo Fisher Scientific (Paisley, United Kingdom). Water (ChromaSolve LC-MS), acetonitrile (HPLC-grade), and formic acid were obtained from Honeywell/Riedel-de Haen (Seelze, Germany) and VWR chemicals (Fontenay sous Bois, France), respectively. All other chemicals were purchased from Sigma-Aldrich (Saint Quentin Fallavier, France) or VWR Chemicals (Fontenay sous Bois, France). Pierce BCA protein Assay kit was purchased from Pierce (Rockford, IL, United States). For all reactions, LoBind Eppendorf tubes (Dutscher, Brumath, France) were used.

### Sample Preparation

#### Brain Protein Extraction Protocol

Lysis buffer containing, trizma-base 20 mM; NaCl 150 mM; cOmplete Protease Inhibitor Cocktail 1X and 1% triton, was added to single pieces of whole brain tissue (∼100 mg) at a ratio of 5 μL per 1 mg of tissue. Brain samples were homogenized by beads beating using a precellys soft tissue CK14 2 mL (3^∗^30S at 6,500 rpm). The lysate was then centrifuged at 4,000 rpm for 15 min at 4°C. Fifty microliters from the supernatant was used for analysis. A 5 μL aliquot was used for total protein concentration, determined by the Pierce BCA Protein Assay kit with a sample to working reagent ratio 1:20. Two percent SDS were added to eliminate interference from lipids.

#### Pellet Digestion

The lysate was precipitated by adding 150 μL of methanol (ratio 3:1), followed by vortex-mixing and briefly centrifuged 5 s. The supernatant was discarded. Twenty microliters of rapigest 0.05% in ammonium bicarbonate 50 mM were added to the pellet. Aqua peptides were added at this step, at 10 ng/mL final concentration. After mixing for approximately 15 min, reduction was performed with 10 μL DTT (20 mM) and incubation at 60°C for 30 min. Alkylation was performed with 10 μL iodoacetamide (45 mM) and 45 min incubation at room temperature. Proteins were digested overnight at 37°C with 40 μg of trypsin.

#### Solid Phase Extraction (SPE)

Tryptic digests were diluted by addition of 300 μL of 5% ammonium hydroxide before SPE extraction on oasis MAX 1 cc/30 mg, previously conditioned with 1 mL of Methanol and equilibrated with 1 mL of water. Samples were loaded and washed with 500 μL of 5% NH40H and 2^∗^250 μL of methanol. Peptides were eluted with 3^∗^250 μL of methanol containing 10% formic acid. Extracts were then evaporated to dryness with a Turbovap instrument (Biotage, Uppsala, Sweden) (5–15 psi, 40°C for 1 h). The dry residue was re-dissolved in 95% water 5% acetonitrile 0.1% formic acid and centrifuged at 4°C for 10 min at 15,000 ×*g*, prior injection into the LC system.

### LC-MS/MS Analysis

LC-MS/MS was performed on a Dionex Ultimate 3,000 chromatography system coupled to a Q-exactive Quadrupole-Orbitrap mass spectrometer (Thermo Fisher Scientific, Bremen, Germany). Ten microliters of sample was loaded onto the column. Chromatographic separation was performed on an Aeris peptide XBC18 reverse phase column (150 mm × 2.1 mm; 1.7 μm; 100 Å; phenomenex, Le Pecq, France) and achieved in a 30 min gradient at a flow rate of 500 μL/min. A gradient of mobile phase consisting of LC-MS-grade water with 0.1% formic acid (phase A) and acetonitrile with 0.1% formic acid (phase B) was delivered. After an isocratic step of 0.5 min at 5% B, the gradient was ramped to 25% over the next 19.5 min then to 50% over the next 4min. Then acetonitrile was increased to 95% for the next 2 min. Column re-equilibration at 5% B was realized for 4 min.

Instrument parameters of the electrospray ionization source were set as follows: sheath gas flow rate at 70 a.u., spray voltage at 4 kV, capillary temperature at 320°C. The Q-exactive instrument was operated in positive ion mode under time-scheduled sequential PRM acquisition. Endogenous peptides precursor ions and AQUA peptides were selected in the quadrupole with an isolation mass window of 1.5 m/z. Precursors were fragmented in the HCD cell using nitrogen as collision gas and the optimized normalized collision energy (**Supplementary Table S2**). All fragment ions were transferred to the Orbitrap. Resolution was set to 70,000 at m/z 200 (full width at half-maximum), automatic gain control to 1e6, and maximum injection time to 240 ms. Xcalibur 2.2 software (Thermo Fisher Scientific, Bremen, Germany) was used for instrument control and processing of the data files.

### PRM and Quantification

A time-scheduled sequential PRM method was established targeting the following C9ORF72 peptides TEIALSGK, ILLEGTER, DSTGSFVLPFR, and SHSVPEEIADIADTVLNDDDIGDSCHEGFLLK (**Supplementary Table S2**). To increase the signal to noise ratio and assay sensitivity, the signal of up to 6 major and non-interfered fragment ions identified with high resolution (5 ppm) from a common peptide precursor were summed up to provide one extracted ion chromatogram (XIC) for each peptide. Isotope-labeled synthetic peptides with labeled amino acids ^13^C_6_,^15^N_2_-labeled lysine and ^13^C_6_,^15^N_4_-labeled arginine were used for signal normalization and quantification of C9ORF72 peptides. Raw MS data were exported to Skyline 3.7 (MacLean et al., 2010) for verification of the transitions ratio of unlabeled and labeled peptides. Xcalibur 2.2 software (Thermo Fisher Scientific, Bremen, Germany) was used for quantitative data analysis.

External calibration curve was made by digesting the C9ORF72 long isoform recombinant protein in a surrogate matrix (mice brain) and SIL peptides were spiked before reduction and alkylation of the pellet. Linear regression with 1/x weighting was applied to generate a standard curve.

### HEK293 Cells Transfection

Plasmids expressing EGFPN-tagged C9ORF72 long or short isoforms were assembled as follow. cDNAs coding for NP_060795 (long) and NP_659442 (short) were ordered from DNA2.0/ATUM in the pCS2 vector. Fifty nanograms of each plasmid was PCR amplified with AccuPrime Pfx Supermix (Invitrogen) according to the manufacturer’s protocol. Forward primer was CACCTCGACTCTTTGCCCACC and reverse primers were, respectively, for the long and short isoforms CTAAAAAGTCATTAGAACATCTCGTTCTTGCACAC and CTACTTGAGAAGAAAGCCTTCATGACAGC. Purified and sequenced PCR products were cloned into pENTR/D Gateway TOPO according to manufacter’s protocol (Thermo Fisher Scientific). Purified and sequenced entry long and short C9ORF72 clones were recombined with pgLAP1 destination vector (Addgene Plasmid #19702) with LR Clonase Enzyme Mix according to the protocol given by the manufacturer (Thermo Fisher Scientific). After sequencing, the two plasmids, EGFP long C9ORF72 and EGFP short C9ORF72, were transfected with lipofectamine 2000 (Thermo Fisher Scientific) according to manufacturer’s protocol. Briefly, T-75 flasks of HEK293 cells at 70% confluency were transfected with 15 μg EGFP long C9ORF72 and 15 μg EGFP plasmids, or 15 μg EGFP short C9ORF72 and 15 μg EGFP plasmids or 15 μg EGFP long C9ORF72 and 15 μg EGFP short C9ORF72 plasmids, or 30 μg EGFP control plasmid. Cells were collected 48 h after transfection. PBS washed pellets were stored at minus 80°C until protein extraction.

### Statistical Analyses

All statistical analysis was performed using Graphpad Prism software (version 5.01). Data were compared with a Mann–Whitney test and medians were considered significantly different if *p* < 0.05. Data were represented with median and interquartile range.

## Results

### Assay Design

**FIGURE 1:**
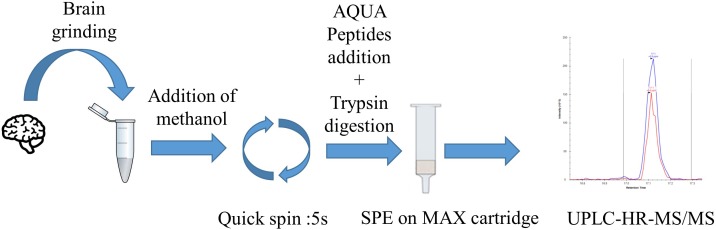
C9ORF72 quantification workflow by pellet digestion and LC-MS/MS in brain samples. First step consists of tissue lysis followed by brain proteins precipitation. Then, the pellet is digested by trypsin. Peptides are extracted and concentrated by SPE before LC-MS/MS injection.

First step of the assay (**Figure 1**) consists in an optimized brain sample homogenization and extraction of C9ORF72 isoforms using bead-beating tubes with the presence of a nonionic surfactant Triton X-100 to further disrupt lipidic cell membranes. Then, a pellet digestion protocol was adapted to protein digestion in brain extracts. C9ORF72 isoforms are denaturated and equivalently precipitated with methanol. After removal of the supernatant containing the surfactant, resuspension and addition of AQUA peptides, i.e., stable isotope peptide, the pellet was digested with trypsin. Signature peptides were then extracted using mixed-mode anion exchange cartridges. Finally, after evaporation and resuspension in 5% acetonitrile with 0.1% formic acid, samples were injected into the LC-MS/MS where three peptides, common to both isoforms or unique to the long isoform, were quantified with specificity in the PRM mode. A specific peptide from the short isoform was also monitored for detection purposes only.

### Analytical Procedure

#### Proteotypic Peptides Selection and LC-MS/MS Detection

**FIGURE 2:**
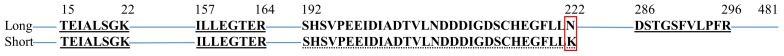
C9ORF72 long (top) and short (bottom) isoform (respectively, Q96LT7-1 and Q96LT7-2). Proteotypic peptids selected are underlined. A red box displays the single amino acid differentiating the common sequence.

Two isoforms of the C9ORF72 protein are reported (DeJesus-Hernandez et al., 2011) (Uniprot sequence entries: Q96LT7-1 and Q96LT7-2). Amino-acid sequences are illustrated in **Figure 2**. Short isoform amino acid sequence is shared with the long one with the exception of its last residue, i.e., residue 222 (N→K). For a comprehensive assay of C9ORF72, common and isoform-specific peptides have to be identified. To this end, data-dependent analysis (DDA) experiments on human frontal cortices were performed (described in Supporting Information). No signal was detected for the C9ORF72 isoforms, similarly, to another recent study (Umoh et al., 2018), illustrating their low abundance in brain and the need to develop a more sensitive targeted method. A tryptic digest of recombinant C9ORF72 long isoform was then used for selection of best responding peptides. Three peptides were selected based on signal intensity after digestion, size (i.e., between 6 and 20 amino acids), and lack of cysteine, methionine and glutamine residues. Specificity of the selected peptides was assessed using Basic Local Alignment Search Tool (BLAST) against the UniprotKB/Swissprot human database. Among the three peptides selected for quantification (**Figure 2**), two are common to both the short and long isoforms and one is unique to the long isoform. The assay based on peptide 1 (TEIALSGK) and 2 (IILEGTER) quantify total C9ORF72 whereas the assay based on peptide 3 (DSTGSFVLPFR) differentiates the long isoform. Internal standards AQUA peptides of the three selected peptides for quantification were synthesized by incorporating stable isotopes at the C-terminal amino acid residues (^13^C_6_,^15^N_2_-labeled lysine and ^13^C_6_,^15^N_4_-labeled arginine). AQUA peptides were prepared with high purity (>95%) and well-defined concentrations. The unique 30 amino acids long peptide specific for the short isoform (peptide 4), containing residue 222 (**Figure 2**), did not meet the stringent selection criteria for inclusion in the quantitative method, and was selected for detection only. A stable isotope-labeled (SIL) analog of peptide 4 was nonetheless synthetized for unambiguous identification of C9-S in the brain samples. In the absence of a suitable quantitative peptide for C9-S, quantification is obtained by difference as previously reported for progastrin-releasing peptide (ProGRP) isoforms (Torsetnes et al., 2013).

**FIGURE 3:**
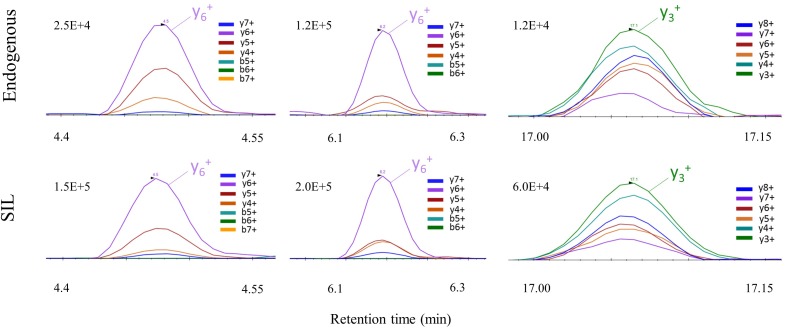
PRM signal of C9ORF72 proteotypic peptides with their corresponding co-eluting SIL peptide in human brain samples.

Simplification of sample preparation oriented our choice toward Parallel Reaction Monitoring (PRM) over Selected Reaction Monitoring (SRM) for the increased specificity regarding fragment ion detection in complex matrixes provided by the high-resolution Orbitrap mass analyzer. A PRM method was established targeting the three selected peptides for quantification, and the unique C9-S peptide for detection only. Ultra-high performance liquid chromatography was performed on a C18 column for peptide separation with a total runtime of 30 min. Shorter gradients resulted in interferences at the retention time of peptide DSTGSFVLPFR (SIL version) and a decrease of signal intensity up to a factor of 2 in brain samples (**Supplementary Figure S1** and **Supplementary Table S1**). Data treatment increased sensitivity by summing the signals of up to six major and non-interfered fragment ions to provide one XIC for each targeted peptide (Dupré et al., 2015; **Figure 3** and **Supplementary Table S2**). Each endogenous peptide and their corresponding isotope-labeled form (AQUA peptides) must strictly co-elute with similar transition ratio across the different samples in comparison with a standard. If not, the transition was excluded.

Ability of the targeted method to detect the isoforms was demonstrated in HEK293 cells transfected with plasmids expressing either C9-S or C9-L. All common (peptides 1 and 2) and the two unique isoform-specific peptides of C9-L and C9-S (peptides 3 and 4) were detected in corresponding HEK293 cells (**Supplementary Figure S3**). Mock-transfected HEK293 cells were also analyzed and displayed a lower level of C9ORF72.

### Sample Preparation of Human Brain Samples Prior to LC-PRM

**FIGURE 4:**
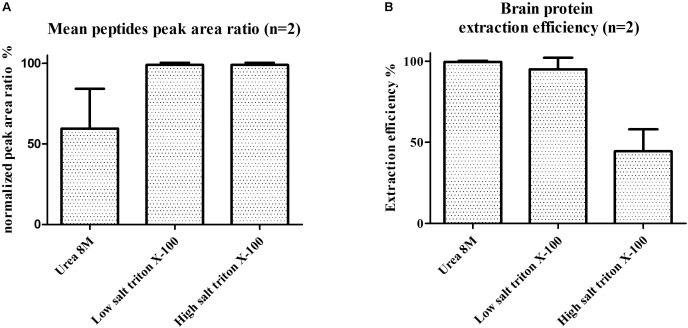
**(A)** Mean C9ORF72 peptides signal in human brain samples and **(B)** brain protein extraction efficiency (measured by BCA assay) depending on tissue lysis protocols. Protocols evaluated contained either 8 M urea, low salt (150 mM NaCl) triton X-100 or high salt (750 mM NaCl) triton X-100.

The main steps of sample preparation consist of tissue homogenization for optimal protein extraction followed by a pellet digestion and peptide enrichment with SPE for lowering ion suppression/matrix effects and increasing sensitivity. Protein extraction protocols with tissue homogenization by mechanical shear recently published for C9ORF72 (Xiao et al., 2015), containing either low/high-salt content (i.e., 150 mM NaCl or 750 mM NaCl), 8 M urea or Triton X-100, were individually evaluated on tissues samples from frontal cortex (**Figure 4**). These protocols were adapted to single use bead-beating Precellys tubes to parallelize protein extraction and avoid contamination between samples. Protocols were evaluated based on total protein extraction and signal from C9ORF72 peptides. The low salt protocol without detergent resulted in low protein extraction yield and no signal for C9ORF72 peptides, although Western blot signal was previously reported for the short isoform (Xiao et al., 2015). Signals were observed for the three quantitative peptides selected for quantification (peptides 1–3) when applying the Triton containing mixtures, the latter showing higher intensity than that containing urea (**Figure 4A**). We finally selected the low-salt Triton protocol because it allowed the extraction of more total proteins than the high-salt Triton protocol (**Figure 4B**). However, no signal was detected for the C9-S unique peptide (peptide 4) with any of the evaluated protocols.

A recently published convenient pellet digestion protocol for monoclonal antibody quantification in human plasma (Becher et al., 2017) was evaluated for the detection and quantification of C9ORF72 isoforms in brain proteins lysates. Recombinant protein of C9-S like and C9-L spiked into mice brain lysate, as a substitute to human brain, before and after methanolic precipitation demonstrated high and equivalent recovery for both recombinant protein isoforms with equivalent precipitation yield above 70% and coefficients of variation below 10% (*n* = 2) (**Table 1**). Brain lysate is a highly complex matrix resulting in deleterious matrix effects. To further reduce its complexity, SPE clean-up was evaluated in human brain samples based on AQUA peptides signal after SPE (**Supplementary Figure S2**). Among Oasis Hydrophilic-Lipophilic Balance (HLB) operated at high pH, Mixed-mode Cation-eXchange (MCX), and Mixed-mode Anion-eXchange (MAX) cartridges, Oasis MAX has proven to give the higher peptide signal intensity (data not shown), in line with the acidic isoelectric point of the peptides ranging from 3.9 to 5.8. Signal enhancement resulting from SPE was about two folds. SPE yield was then determined in the final conditions in mice brain lysate. Yield measured by aqua peptides spiked before and after the SPE extraction were between 45 and 70% (**Supplementary Table S3**), which is in line with previous works (Gong et al., 2015). For method robustness, aqua peptides were spiked early in the protocol, before trypsin digestion of the pellet, and therefore extracted by SPE, similarly, to the C9ORF72 peptides.

**Table 1:** Precipitation yield and variation of C9-S and C9-L isoforms in mice brain extracts.

	Short isoform		Long isoform	
	Mean	CV	Mean	CV
Precipitation yield (*n* = 2)	88%	8%	74%	9%

### Method Validation

The assay was then evaluated for C9ORF72 quantification in human brain tissues, including the main items of linearity, sensitivity, inter-, and intra-day assay precision, stability during sample processing, i.e., over 90 min at room temperature and matrix effect. Linearity and sensitivity were determined with standard curves prepared in mice brain as a surrogate to human brain samples. Indeed, the three proteotypics peptides selected for quantification are not present in mice due to single point mutations, despite 85% sequence identity with human (**Supplementary Figure S4**). Other items were tested with quality control samples prepared in human brain extract, spiked with recombinant C9-L. Recombinant C9-L was selected for validation experiments because it contains all three quantitative peptides.

**FIGURE 5:**
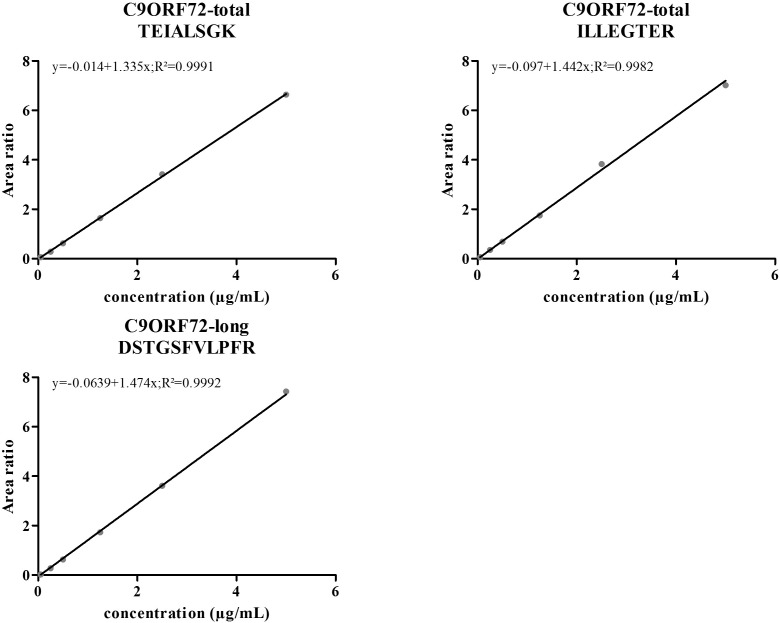
Standard curves of the three selected peptides for quantification of human C9ORF72 in a mice brain lysate. C9ORF72 recombinant long isoform was spiked in increasing concentrations, allowing the generation of a six points standard curves (weighing 1/x).

**FIGURE 6:**
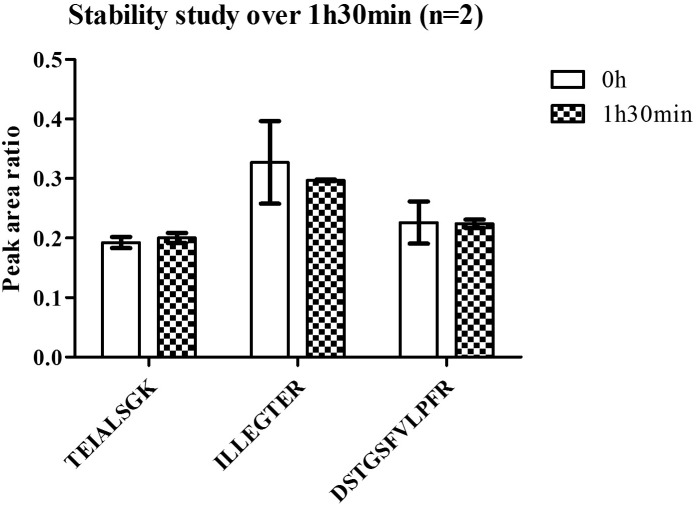
Stability of C9ORF72 in human brain at room temperature.

Linearity and sensitivity of the method were evaluated with a 6-point calibration curve of the recombinant C9-L. Mice brain proteins were extracted with the same protocol as for human brain. The method was shown to be linear from 50 to 5,000 ng/mL with a Lower Limit Of Quantification (LLOQ) observed at 50 ng/mL (**Figure 5**). LLOQ was defined based on accuracy between 80 and 120%. To determine a potential matrix effect between mice and human brains, recombinant C9ORF72 was spiked into human brain extract in triplicates at 500 ng/mL and confronted against the calibration curve in mice (**Table 2**). The three peptides displayed good accuracy in the range of 85–115%, which demonstrate the suitability of mice brain extract as a surrogate matrix for C9ORF72 determination in human brain. Intraday repeatability of the analytical method was evaluated in human brain extract. Briefly, proteins from a human brain sample were extracted and divided in five aliquots for protein precipitation, digestion and LC-MS/MS analysis. Precision was observed below 10% for each peptide (*n* = 5) (**Table 3**). Interday precision was also evaluated by analyzing three different brain extracts on three different days. Variability was found to be acceptable, with CV% between 10 and 26% (**Table 4**). Stability of the C9ORF72 protein in brain extract during the sample preparation is an important parameter for quantification. Brain extracts were either directly processed or left on ice for 90 min, corresponding to the time to process around 100 samples from protein extraction to protein precipitation. Both conditions showed similar area ratio for the three peptides demonstrating stability of C9ORF72 in our conditions (**Figure 6**).

**Table 2:** Matrix effect between mice and human brain, evaluated for each quantitative peptide.

QC in human brain (*n* = 3)	TEIALSGK				ILLEGTER				DSTGSFVLPFR			
	Mean measured (μg/mL)	CV %	Theoretical value (μg/mL)	Bias%	Mean Measured (μg/mL)	CV%	Theoretical value (μg/mL)	Bias %	Mean measured (μg/mL)	CV %	Theoretical value (μg/mL)	Bias %
Blank (endogenous)	0.285	6%			0.292	2%			0.348	5%		
Spike (+0.5 μg/mL)	0.767	4%	0.785	2%	0.815	1%	0.792	3%	0.785	3%	0.848	7%

Human brain lysate was spiked with 0.5 μg/mL of C9ORF72 recombinant long isoform and back calculated against a calibration curve prepared in mice brain lysate. Endogenous C9ORF72 concentration was added to the spiked amount for calculations of bias. Bias and CV% are reported.

**Table 3:** Intraday variability of the measured C9ORF72 concentrations by replicate analysis of a control human brain sample (*n* = 5).

Intraday validation (*n* = 5)	TEIALSGK		ILLEGTER		DSTGSFVLPFR	
	Mean (μg/mL)	CV	Mean (μg/mL)	CV	Mean (μg/mL)	CV
Control-1	0.37	4%	0.43	7%	0.414	6%


**Table 4:** Interday variation of the measured C9ORF72 concentrations in 2 C9ORF72 human brains and one human control brain samples; (*n* = 3).

Interday variation (*n* = 3)	TEIALSGK		ILLEGTER		DSTGSFVLPFR	
	Mean (μg/mL)	CV	Mean (μg/mL)	CV	Mean (μg/mL)	CV
Control-2	0.514	18%	0.545	10%	0.563	14%
C9-1	0.344	26%	0.382	12%	0.390	22%
C9-2	0.153	11%	0.182	16%	0.197	10%


Taking all results together, the protocol was found efficient for determination of C9ORF72 in human brain samples. The new method demonstrated robustness with variability and accuracy below 20%.

### C9ORF72 Isoforms Determination in Human Brain Tissue

Levels of C9ORF72 protein were investigated in frontal cortices of FTD patients (with or without ALS) carrying *C9ORF72* expansions (*n* = 21), patients with non-genetic FTD, pathologically characterized by TDP-43-positive neuronal inclusions (*n* = 10), and neurologically normal controls (*n* = 12) determined by pathologists within the network of the NeuroCeb brain bank.

**FIGURE 7:**
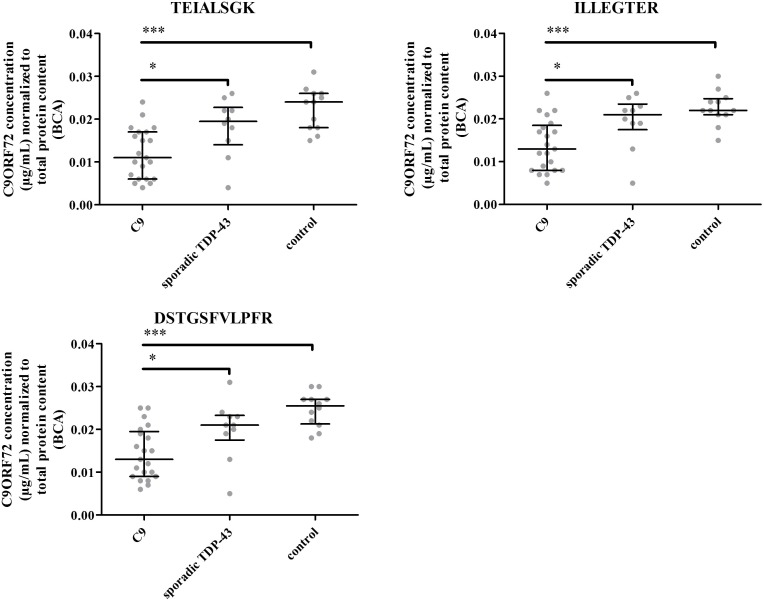
C9ORF72 concentration determination based on peptides 1, 2, and 3 in the human brain frontal cortex cohort. ^∗^*P* ≤ 0.05 and ^∗∗∗^*P* ≤ 0.001.

The three peptides selected for quantification were detected above LLOQ in all samples (**Figure 7**). However, no signal was detected for the unique C9-S peptide in any of the brain samples. Furthermore, similar amounts of total C9ORF72 and C9-L were measured, considering assay accuracy and variability of 20%, revealing by difference, the low abundance of C9-S (**Supplementary Table S4**). Differences in measured concentrations between groups were assessed by a Mann–Whitney nonparametetric test. Quantification based on any of the three peptides demonstrated a significant decrease in total C9ORF72 in FTD patient with *C9ORF72* expansions (*p* < 0.0001) compared to controls and non-genetic FTD (*p* < 0.05) (**Figure 7**). The decrease in concentration of about 42%, which was observed for the three peptides (i.e., the two peptides representing total C9ORF72 and the one specific to the long isoform), corroborates prior Western blot findings.

## Discussion

C9ORF72 isoforms have solely been investigated by Western blot which relies on the availability of antibodies whose specificity has to be characterized and validated (Liu et al., 2006; Davidson et al., 2018). In this respect, we developed a MS assay avoiding antibodies and the gold standard immunoprecipitation for protein quantification in complex matrixes. An efficient protocol, with optimized sample preparation steps, i.e., protein extractions from tissue, pellet digestion and SPE, was successfully implemented for the first time to quantitate C9ORF72 isoforms level in a cohort of human brain samples from *C9ORF72* or non-genetic FTD patients and control individuals.

Preparation of tissue lysates and protein extraction is a key issue for proteome coverage, especially the more challenging membrane or nuclear proteins (Cox and Emili, 2006; Wiśniewski et al., 2009). Taking into account the reported distinct subcellular localizations of C9ORF72 isoforms with localization of C9-S to the nuclear membrane and C9-L in the cytoplasm (Xiao et al., 2015), lysis protocols were investigated. We found that Triton X-100 facilitated the extraction of the C9ORF72 proteins, in agreement with higher extraction yield reported with detergent based protocols in comparison to organic solvents or chaotropic reagents such as urea, in fatty tissue such as the brain (Shevchenko et al., 2012). In addition, the Triton X-100 protein extraction protocol probably enhanced the subsequent trypsin digestion as previously reported (An et al., 2015). A simple pellet digestion protocol (Becher et al., 2017) was used here for removal of potentially interfering matrix components such as small molecules, phospholipids, peptides (Ouyang et al., 2012) and the added Triton X-100 surfactant which could otherwise have a dramatic impact on MS sensitivity (Cox and Emili, 2006). Considering the molecular weight difference between C9-S and C9-L, i.e., 25 and 54 kDa, and the potential solubility of smaller proteins in organic precipitation solvents (Lopes et al., 2014), we ascertained the equivalent recovery for both isoforms, ensuring accurate quantification. The signal observed for both C9-S and C9-L unique peptides in transfected HEK293 cells further confirmed the correct extraction and precipitation of C9ORF72 isoforms.

The validation experiments demonstrated that the precision provided by the method was satisfactory, with coefficients of variation below 20% and its ability to determine C9ORF72 relative concentration in human brain samples with a LLOQ at 50 ng/mL. Prior work with pellet digestion reported LLOQ around 1 μg/mL for quantification of therapeutic antibodies in plasma (Ouyang et al., 2012; Becher et al., 2017). In the present work, the use of PRM mode for peptide detection offered additional selectivity linked to the high resolution and mass precision measurements in the Orbitrap and the opportunity to accumulate fragment ions in the C-trap, eventually summed-up for higher signal intensity. Regarding signature peptide selection, chimeric or humanized therapeutic antibodies are more and more alike and so only a few peptides are unique to one therapeutic antibody. These unique peptides do not necessarily have the best physicochemical properties to be efficiently ionized by electrospray ionization whereas C9ORF72 peptides were selected based on signal intensity. Also, the fivefold lower protein content in brain extract found by BCA may have contributed to reduced deleterious matrix effects compared to plasma. Most probably, a combination of these factors explains the gain in sensibility obtained for C9ORF72.

So far, C9ORF72 quantification was only accomplished by Western blot using in-house generated antibodies. The new MS assay allowed for the first time monitoring of C9ORF72 isoforms in human brain samples, without potential interference due to cross-reactivity of antibodies. First, we observed equivalent brain levels for C9-total and C9-L, indicating a low abundance of C9-S within the assay variability, i.e., a concentration below 20% of C9-L. Here, a similar peptide release between the recombinant and endogenous C9-S/C9-L was assumed since the protocol denature proteins during tissus lysis and pellet digestion which enhances digestion by protein unfolding (Pritchard et al., 2014). The low abundance of C9-S is further strenghtened by the undetected unique C9-S peptide regardless of the evaluated brain protein extraction protocol, including those previously published for C9ORF72 (Xiao et al., 2015), whereas the peptide was well detectable in transfected HEK293 cells. Considering the lower abundance of the short isoform, deeper fractionation of the sample, for instance through enrichment of gray matter or subcellular fractions, could be considered to increase assay sensitivity. Also, post-translational modifications could impact peptide detection by our targeted method, even though none was reported for any of the selected peptides by now. In previous Western blot reports, Waite et al. (Waite et al., 2014) found that C9-S was in lower abundance than C9-L but questioned the specificity of their antibody, two bands being present at 27 and 29 kDa. Although, Xiao et al. (2015) detected the C9-S in frontal cortex using isoform specific antibodies, the relative abundance between C9-L and C9-S was not determined, probably in relation to the inherent limitation of protein quantification by Western blotting which depends on the affinity and specificity of the reagents (Aebersold et al., 2013). Next, we were able to confirm in a collection of 43 frontal cortices the diminution of the C9ORF72 long protein concentration in *C9ORF72* FTD patients corroborating previous observations by the two Westernblot studies (Waite et al., 2014; Xiao et al., 2015) and at the mRNA level. The age of onset of the disease is highly variable as well as the number of expansion, the precise determination of C9ORF72 levels afforded by the new assay can be used to investigate correlations between the length of expansion and the levels of C9ORF72.

In summary, an efficient protocol was developed for quantification of C9ORF72 isoforms in brain samples by MS. Combination of optimal sample preparation and targeted high-resolution MS demonstrated robust and efficient quantification ability. This new assay has the advantage of being based on MS, avoiding the potential cross-reactivity of antibodies and simplifying implementation in various laboratories (Addona et al., 2009). C9ORF72 long isoform was significantly decreased in carriers of *C9ORF72* expansion in comparison with controls and non-genetic FTD patients with or without ALS, corroborating prior observations made by Western blot and at the mRNA level. Whereas some studies reported a short isoform, here it represents less than 20% of the long one, suggesting possible non-specificity or cross-reactivity of antibodies. To our knowledge, this is the first report of a MS-based quantification assay for C9ORF72 proteins. This method needs to be further applied to other biological matrixes of a more relevant diagnostic nature and potentially to follow treatment efficacy in the future. This method could be easily implemented to mice models of *C9ORF72* FTD or other animal model owing to a highly conserved sequence, in order to advance understanding of the contribution of C9ORF72 to disease mechanisms.

## Author Contributions

AV, CF, ILB, FL, VA, and FB contributed conception and design of the study. AV, CF, AC, ML, and VA conducted the experiments. NeuroCEB brain Bank provided samples. AV and FB wrote the first draft of the manuscript. ILB, CF, and VA wrote sections of the manuscript. CF, FF, FE, ILB, CJ, and FL critically reviewed the manuscript. All authors contributed to manuscript revision, read, and approved the submitted version.

## Conflict of Interest Statement

The authors declare that the research was conducted in the absence of any commercial or financial relationships that could be construed as a potential conflict of interest. The handling Editor declared a shared affiliation, though no other collaboration, with one of the authors FE.
