# Supplementary material for: New Antibody-Free Mass Spectrometry-Based Quantification Reveals That C9ORF72 Long Protein Isoform Is Reduced in the Frontal Cortex of Hexanucleotide-Repeat Expansion Carriers
Source: Front Neurosci. 2018 Aug 28;12:589. doi: 10.3389/fnins.2018.00589 (PMC6122177; doi:10.3389/fnins.2018.00589)
Supplement: Supplementary file 1 [file Table_1.docx]

**SUPPORTING INFORMATION**

**New antibody-free mass spectrometry-based quantification reveals that C9ORF72 long protein isoform is reduced in the frontal cortex of hexanucleotide-repeat expansion carriers.**

Arthur Viodé^1^, Clemence Fournier^2,6^, Agnès Camuzat^2,3^, François Fenaille^1^, NeuroCEB brain bank, Morwena Latouche^3^, Fanny Elahi^4^, Isabelle Le Ber^2,5,6^_,_ Christophe Junot^1^, Foudil Lamari^7,8^, &Vincent Anquetil^2,6^, François Becher^1*^

1. Service de Pharmacologie et Immunoanalyse (SPI), Laboratoire d’Etude du Métabolisme des Médicaments (LEMM), CEA, INRA, Université Paris Saclay, F-91191 Gif-sur-Yvette cedex, France.
2. Institut du Cerveau et de la Moelle épinière, ICM, Inserm U 1127, CNRS UMR 7225, Sorbonne Université, F-75013, Paris, France.
3. Ecole Pratique des Hautes Etudes - EPHE, PSL Research University, 75014, Paris, France.
4. Memory and Aging Center, Department of Neurology, University of California, San Francisco,

675 Nelson Rising Lane, Suite 190, San Francisco, California 94158, USA

1. Institute of Memory and Alzheimer’s Disease (IM2A), Centre of excellence of neurodegenerative disease (CoEN), ICM, APHP Department of Neurology, Hopital Pitié-Salpêtrière, University Paris 6, Paris, France.
2. Assistance Publique – Hôpitaux de Paris, Hôpital Pitié-Salpêtrière, Centre de référence Démences Rares, F-75013, Paris, France.
3. Assistance Publique – Hôpitaux de Paris Hôpitaux universitaires Pitié-Salpêtrière-Charles-Foix Service de Biochimie Métabolique, 75013, Paris, France.
4. GRC 13 Neurométabolisme – UPMC,Sorbonne Université, F-75013, Paris, France

**Table of content:**

Supplementary Method 2

Table S1 3

Table S2 4

Table S3 5

Table S4 6

Figure S1 7

Figure S2. 8

Figure S3. 9

Figure S4. 10

Supplementary Method**: C9ORF72 peptides identification by Data-Dependent Acquisition**

The identification of C9ORF72 peptides was done manually. An in-silico-digest of C9orf72 isoforms was performed. The m/z ratio of the resulting peptides was searched within the full MS scan following data-dependent acquisition (top 5 ions) on the Q-Exactive. Peptides were assumed as identified, if the m/z ratio of the parent measured in the Orbitrap was within 5 ppm to the theoretical m/z ratio, and if at least 3 fragment ions from the corresponding MS/MS spectrum could be assigned. The same method was applied to a brain sample using database search with Proteome Discoverer 1.3 (Thermo Scientific) and SEQUEST.

**Supplementary Tables and Figures**

Table S1**.** Effect of gradient length on the peak area of targeted peptides of endogenous C9ORF72 in a human brain sample.

|  | **TEIALSGK** | | **ILLEGTER** | | **DSTGSFVLPFR** | |
| --- | --- | --- | --- | --- | --- | --- |
|  | Peak area (a.u.) |  | Peak area (a.u.) |  | Peak area (a.u) |  |
| Short-gradient  (15 min total run time) | 123403 |  | 68044 |  | 142012 |  |
| Long-gradient (30 min total run time) | 159265 |  | 124394 |  | 195284 |  |
| Gain (long vs. short) | 30% |  | 80% |  | 40% |  |

.

| Peptide sequence | Average mass (Da) | Precursor ion m/z | Product ion m/z* | Normalized collision energy % | Retention time (min) |
| --- | --- | --- | --- | --- | --- |
| TEIALSGK | 817.94 | 409.9 (z=2) | 404.2504 (y4) | 17 | 4.5 |
|  |  |  | 475.2875 (y5) |  |  |
|  |  |  | 588.3715 (y6) |  |  |
|  |  |  | 717.4141 (y7) |  |  |
| TEIALSGK[^13^C_6_; ^15^N_2_] | 825.88 | 413.74 (z=2) | 412.2646 (y4) | 17 | 4.5 |
|  |  |  | 483.3017 (y5) |  |  |
|  |  |  | 596.3857 (y6) |  |  |
|  |  |  | 725.4283 (y7) |  |  |
| IILEGTER | 930.08 | 465.97 (z=2) | 462.2307 (y4) | 17 | 6.2 |
|  |  |  | 591.2733 (y5) |  |  |
|  |  |  | 704.3573 (y6) |  |  |
| IILEGTER[^13^C_6_; ^15^N_4_] | 940.00 | 470.77 (z=2) | 472.2390 (y4) | 17 | 6.2 |
|  |  |  | 601.2815 (y5) |  |  |
|  |  |  | 714.3656 (y6) |  |  |
| DSTGSFVLPFR | 1225.37 | 613.31 (z=2) | 419.2401 (y3) | 20 | 17.1 |
|  |  |  | 532.3242 (y4) |  |  |
|  |  |  | 631.3926 (y5) |  |  |
|  |  |  | 778.4610 (y6) |  |  |
|  |  |  | 865.4930 (y7) |  |  |
|  |  |  | 922.5145 (y8) |  |  |
| DSTGSFVLPFR[^13^C_6_; ^15^N_4_] | 1235.3 | 618.32 (z=2) | 429.2484 (y3) | 20 | 17.1 |
|  |  |  | 542.3325 (y4) |  |  |
|  |  |  | 641.4009 (y5) |  |  |
|  |  |  | 455.4693 (y6) |  |  |
|  |  |  | 875.5013 (y7) |  |  |
|  |  |  | 932.5228 (y8) |  |  |
| SHSVPEEIDIADTVLNDDDIGDSCHEGFLLK | 3441.7 | 861.3 (z=4) | 917.8992 (y16 +2) | 20 | 20.7 |
|  |  |  | 974.4413 (y17 +2) |  |  |
|  |  |  | 1132.0128 (y20 +2) |  |  |
|  |  |  | 1167.5314 (y21 +2) |  |  |
| SHSVPEEIDIADTVLNDDDIGDSCHEGFLLK[^13^C_6_; ^15^N_2_] | 3449.7 | 863.15 (z=4) | 921.9063 (y16 +2) | 20 | 20.7 |
|  |  |  | 978.4484 (y17 +2) |  |  |
|  |  |  | 1136.0199 (y20 +2) |  |  |
|  |  |  | 1171.5385 (y21 +2) |  |  |

Table S2. MS parameters for PRM detection of C9ORF72 peptides as well as corresponding SIL internal standards

Table S3. SPE extraction recovery and CV% measured for each quantitative peptide.

|  | **TEIALSGK** | | **ILLEGTER** | | **DSTGSFVLPFR** | |
| --- | --- | --- | --- | --- | --- | --- |
|  | Mean | CV | Mean | CV | Mean | CV |
| Recovery | 68% | 7% | 45% | 3% | 70% | 16% |

Table S4. Mean difference between C9ORF72 protein concentrations obtained by peptides 1 and 2, peptides 1 and 3, peptides 2 and 3, normalized to the mean total concentration (mean of concentrations determined by peptides 1 and 2).

| **Patient**   **classification** | **Pep 1 – Pep 2** | **Pep 1 – Pep 3** | **Pep 2- Pep 3** |
| --- | --- | --- | --- |
| C9 | 14% | 16% | 2% |
| TDP-43 | 6% | 10% | 4% |
| Control | 1% | 11% | 10% |
