# Supplementary figures and images for: New Antibody-Free Mass Spectrometry-Based Quantification Reveals That C9ORF72 Long Protein Isoform Is Reduced in the Frontal Cortex of Hexanucleotide-Repeat Expansion Carriers
Source: Front Neurosci. 2018 Aug 28;12:589. doi: 10.3389/fnins.2018.00589 (PMC6122177; doi:10.3389/fnins.2018.00589)

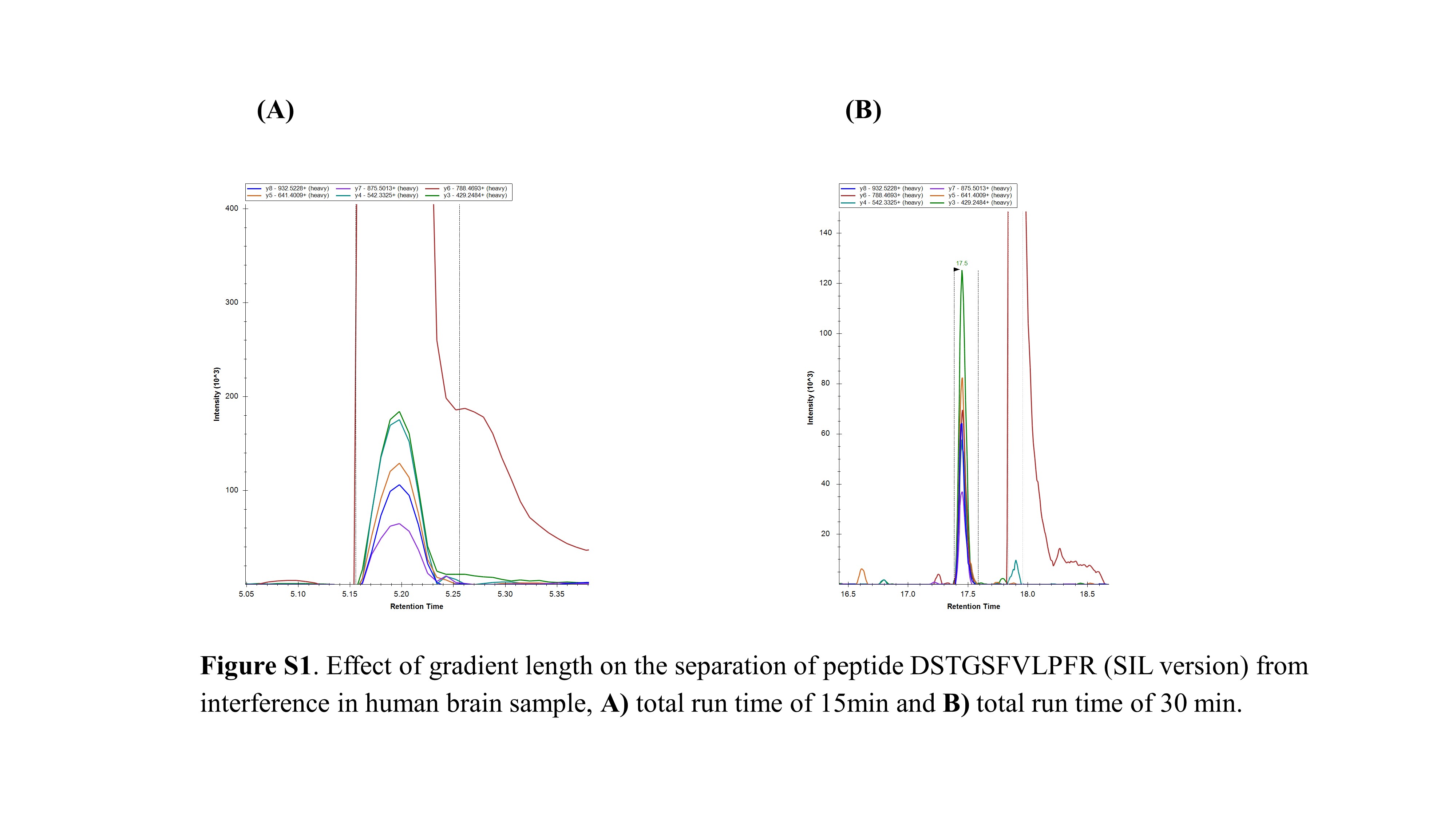

Supplement: Supplementary file 2 [file Image_1.JPEG]

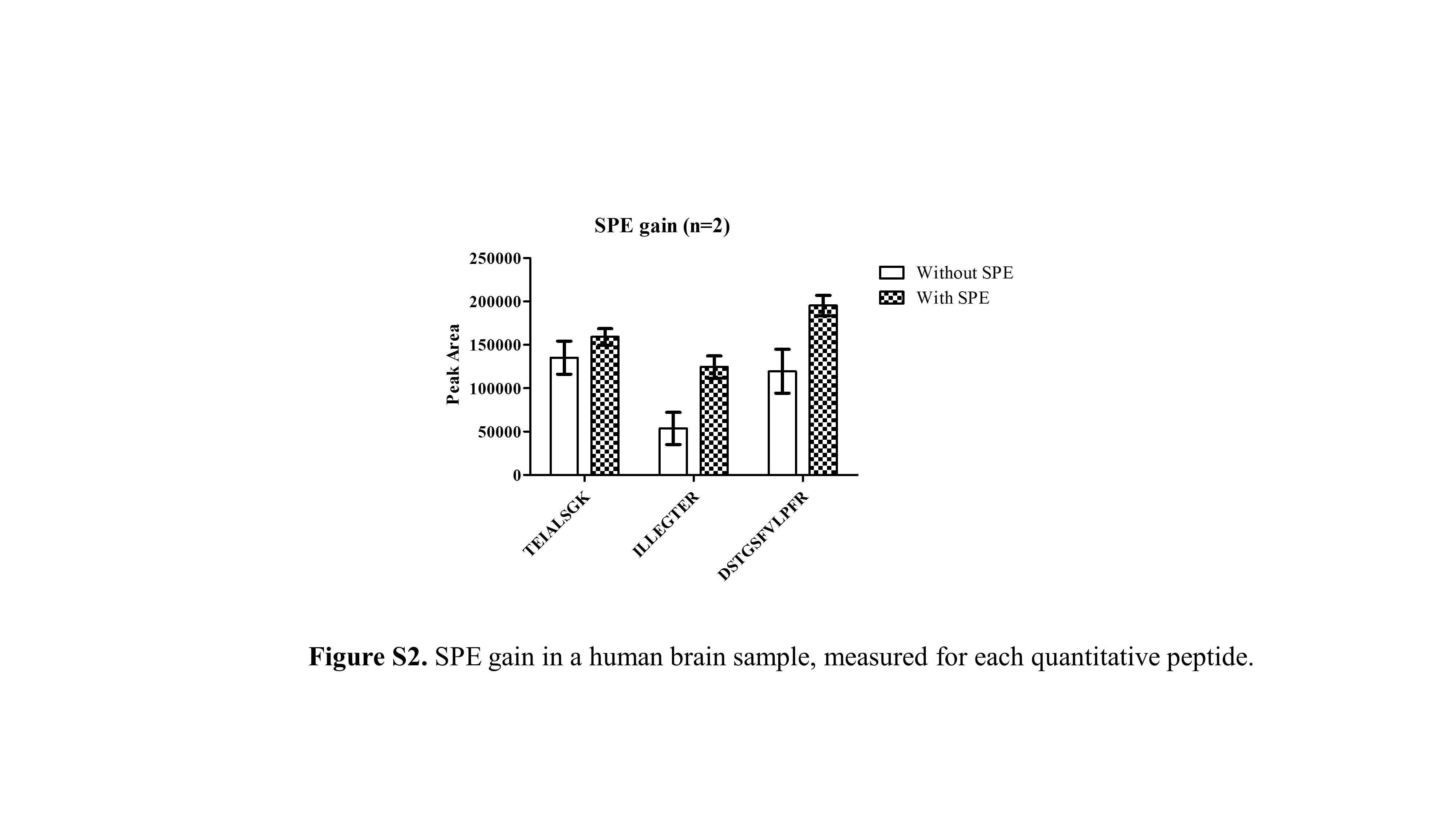

Supplement: Supplementary file 3 [file Image_2.JPEG]

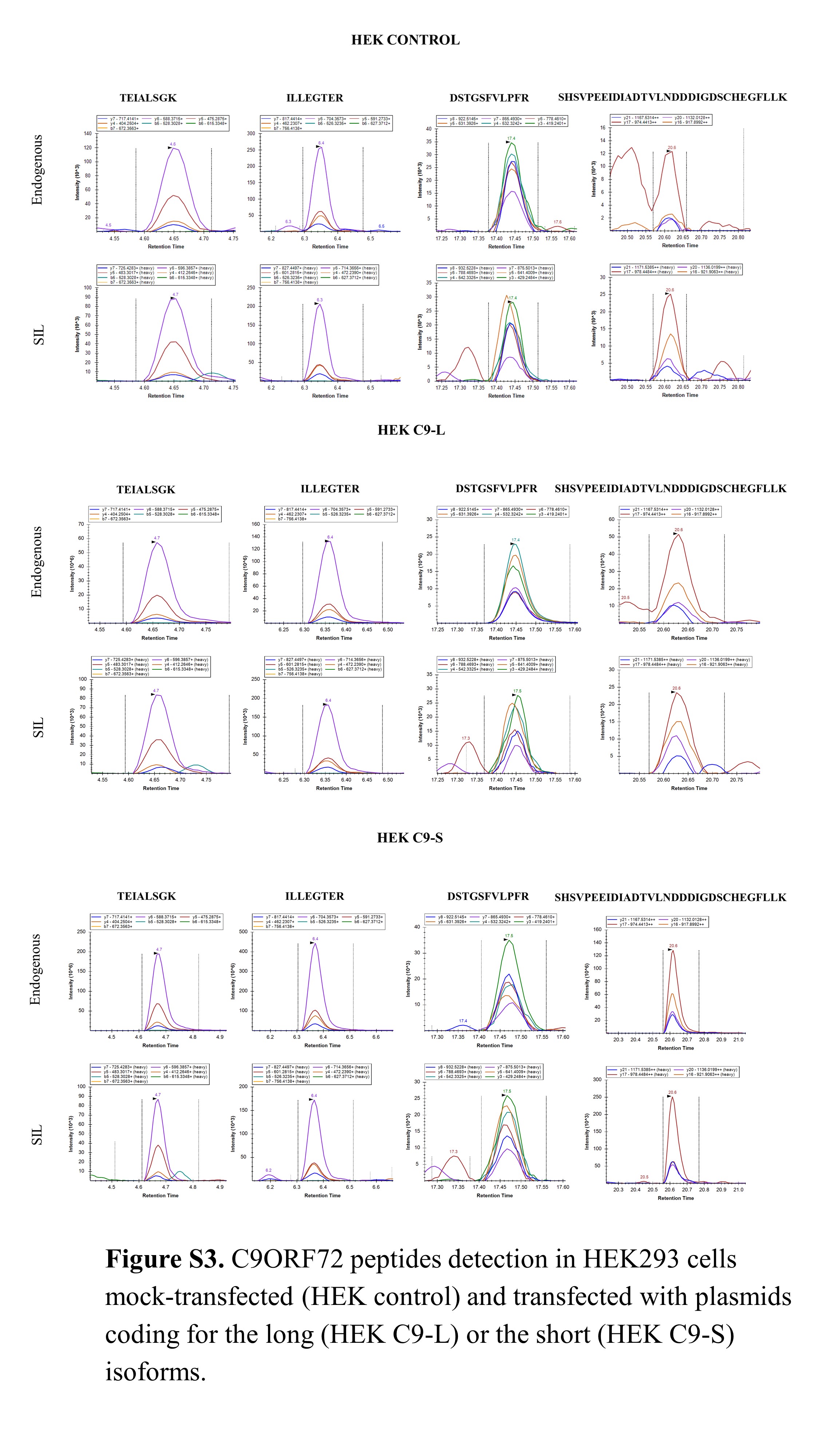

Supplement: Supplementary file 4 [file Image_3.jpg]

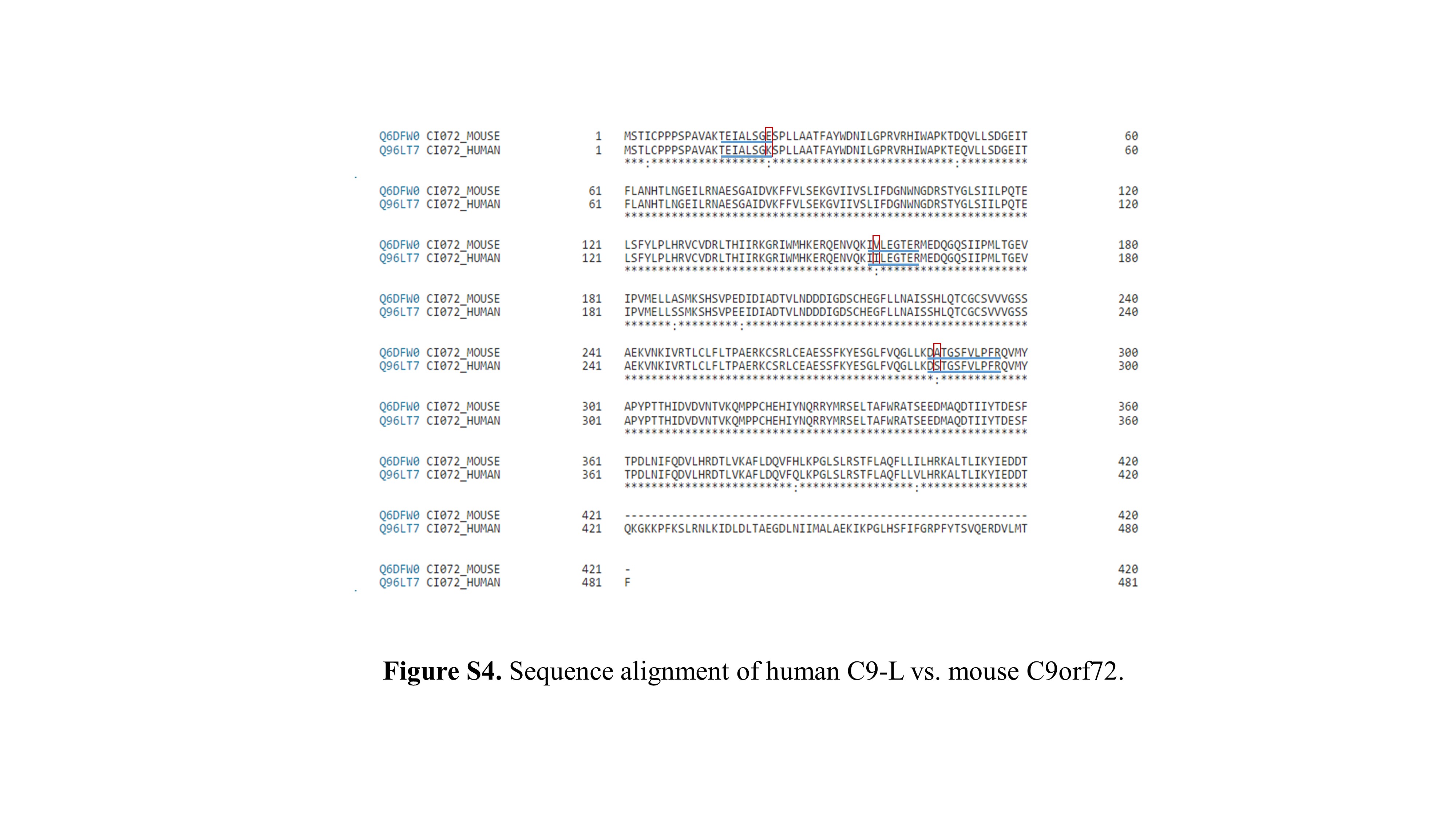

Supplement: Supplementary file 5 [file Image_4.JPEG]
